# Development and functional adaptation of intestinal macrophages across the lifespan

**DOI:** 10.1002/cti2.70111

**Published:** 2026-06-16

**Authors:** Baha Mustafa, Elisa L Hill‐Yardin, Sarah J Spencer

**Affiliations:** ^1^ School of Health and Biomedical Sciences RMIT University Bundoora Melbourne VIC Australia

**Keywords:** ageing, embryonic‐derived macrophages, gastrointestinal development, inflammation, monocyte‐derived macrophages, ontogeny

## Abstract

Macrophages of the gastrointestinal system are central regulators of gut development, homeostasis and disease, yet their origin, functional diversification and life stage‐specific roles remain incompletely integrated. This review aimed to provide a comprehensive synthesis of current knowledge on intestinal macrophage ontogeny, heterogeneity and function from prenatal development through adulthood and into the aging phase. We highlight emerging evidence defining embryonic and monocyte‐derived macrophage populations, their specialised roles in tissue remodelling, immune regulation, vascular and neural support, and their dynamic turnover across the lifespan. The review also examines how disruption of key regulatory pathways, including those relating to interleukin 10 (IL10), transforming growth factor (TGFβ) and metabolic signalling, contributes to macrophage dysfunction in inflammatory bowel disease, as an example of a gastrointestinal disorder with macrophage involvement. By integrating findings from lineage‐tracing, single‐cell transcriptomics and functional studies, this review provides a unified framework for understanding intestinal macrophage biology across life stages. This review provides a strengthened understanding of intestinal macrophage biology and establishes a knowledge base for translational therapies that can modify macrophage function to target inflammatory disorders and maintain gut health.

## Graphical abstract

This article discusses intestinal macrophage development, including the influence of developmental origins and life stages; ontogeny and functional heterogeneity; preclinical and clinical evidence.

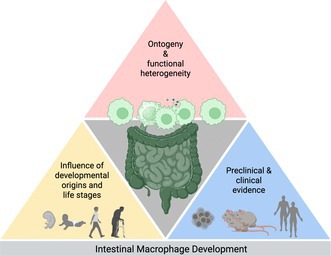

## Introduction

The mammalian gastrointestinal tract is continually exposed to a diverse array of foreign antigens, leading to the presence of the largest population of mononuclear phagocytes (MPs) within the body.^1^, ^2^ This MP population includes monocytes, macrophages and dendritic cells, with macrophages being the most abundant.^1^, ^2^ Macrophages are large immune cells, characterised by a kidney‐shaped or oval nucleus and an irregular structure that enhances their migration and phagocytic abilities. Macrophages play critical roles as primary responders to pathogen invasion^3^ and in maintaining intestinal homeostasis.^4^, ^5^ Emerging evidence also suggests that macrophages influence the development of intestinal tissues^6^ and can acquire specific phenotypes and functions based on the surrounding microenvironment.^4^, ^7^ The origin of macrophages in various organs has been extensively studied,^8^, ^9^, ^10^, ^11^ revealing that these cells can arise from either embryonic progenitor cells or haematopoietic stem cells. Based on early fate‐mapping studies in mice, it was proposed that intestinal macrophages originate from embryonic progenitors during development,^7^, ^9^, ^10^, ^12^ eventually being replaced by monocyte‐derived macrophages.^12^, ^13^, ^14^, ^15^ However, a long‐lived macrophage population has also been identified in adult intestinal tissues.^7^, ^10^, ^12^, ^13^ Nevertheless, it remains unclear whether these two macrophage populations have distinct roles in the maintenance of intestinal homeostasis. Furthermore, the origin, functional diversification and life stage‐specific roles of macrophages remain incompletely described and their sex‐specific roles and functions during aging are not well‐defined. Specifically, we do not yet understand how macrophage development changes at specific stages of intestinal development or the mechanisms that regulate macrophage turnover at the various life stages, the impact of biological sex, biological age. In this review, we address these gaps by focussing on how intestinal macrophages develop, diversify and adapt during distinct life stages. By integrating findings from both animal models and human studies, we aim to provide a life‐course framework that places macrophage ontogeny and heterogeneity in context and highlights key areas where further research is needed to understand age‐specific changes in intestinal immunity and how they relate to clinical disease states.

## Intestinal macrophages in the prenatal period

**Figure 1:**
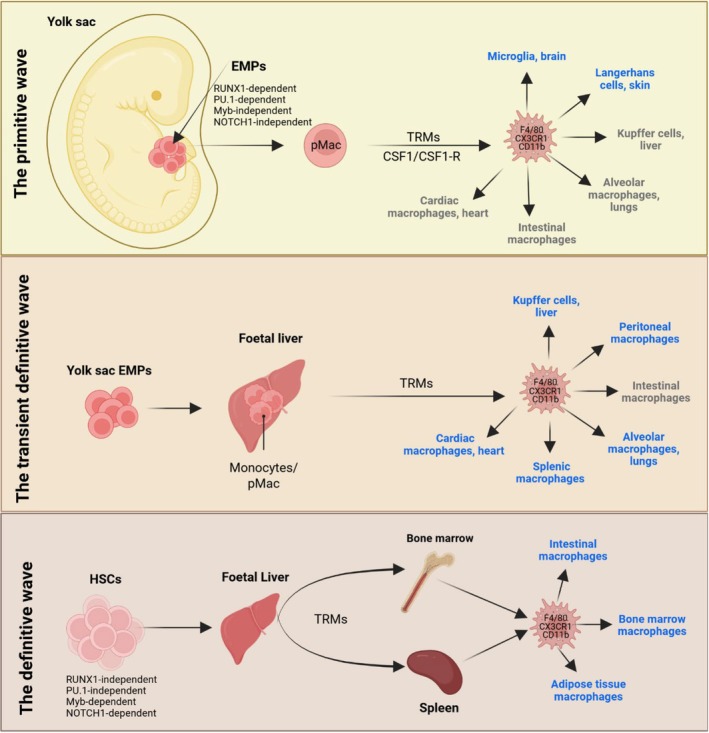
Tissue‐resident macrophages develop in three waves. The primitive wave involves yolk sac‐derived erythro‐myeloid progenitors (EMPs) producing primitive macrophages (pMacs) that migrate to embryonic tissues. In the transitional definitive wave, EMPs enter the foetal liver, differentiate into monocytes, and travel via the bloodstream to become tissue‐resident macrophages (TRMs). The definitive wave involves haematopoietic stem cells (HSCs) from the aorta‐gonad‐mesonephros region migrating to the foetal liver, seeding the bone marrow and generating monocytes that circulate, colonise organs and differentiate into TRMs. Blue text indicates cells with major contributions from the specified wave, while grey text represents cells with minor contributions during adulthood. Created with BioRender.com; Toronto, Canada.

During foetal development, macrophages predominantly originate from yolk sac and foetal liver erythro‐myeloid progenitors (EMP, Figure 1).^8^, ^16^, ^17^ These ‘embryonically derived macrophages’ (i.e. derived from embryonic‐progenitor cells) seed the intestinal tissue *in utero*, establishing the initial macrophage compartment, which is progressively supplemented and largely replaced after birth by monocytes derived from definitive haematopoietic stem cells in the bone marrow.^12^, ^13^, ^14^, ^15^

Anatomically, the intestine consists of two major compartments: the mucosa and the muscularis externa. The mucosa includes the epithelial layer, lamina propria (Lp) and submucosa. Tissue‐resident macrophages are enriched within the Lp of the mucosa, but are also detected in deeper compartments, including the muscularis externa. In mice, ionised calcium‐binding adapter molecule 1 (IBA1)‐positive macrophages colonise the mucosa and submucosa of the mid‐hindgut as early as embryonic day (E) 10.5.^6^ IBA1^+^ macrophages are also present in the outer gut wall at E9.5 (Table 1), coinciding with the emergence of the muscularis layers and the initial invasion of enteric neural crest‐derived cells.^18^ In humans, intestinal macrophages have traditionally been reported at 11–12 weeks of gestation^19^, ^20^; however, single‐cell sequencing recently identified a distinct macrophage cluster in the proximal foetal intestine as early as 7 weeks, indicating that colonisation may commence earlier than previously appreciated.^6^, ^21^ Foetal intestinal macrophages require colony‐stimulating factor 1 (CSF1) and CSF1 receptor (CSF1R) signalling for their development, survival and long‐term maintenance.^22^, ^23^

**Table 1:** Summary of murine studies examining the ontogeny of intestinal macrophage populations across various life stages and under steady‐state conditions

Study	Region	Layer	Age/life stage	Main findings
Bain *et al*.	Colon	Mucosa	Prenatal: E19.5 Neonatal: 2–14 days Adult: 35–63 days	Two populations were identified: F4/80^hi CD^11b^lo^ embryonically derived macrophages, predominant in prenatal and neonatal stages and ~20% in adults, and F4/80^lo^ CD11b^hi^ monocyte‐derived macrophages, which dominate in adults (~80%)
De Schepper *et al*.	Small intestine	Lamina propria & muscularis externa	20–35 weeks	In adult mice, most CD64^+^ macrophages were monocyte‐derived, although ~20–28% of muscularis externa and ~8% of lamina propria macrophages remained un‐replaced, suggesting the presence of long‐lived, self‐maintaining macrophages
Shaw *et al*.	Colon & small intestine	Lamina propria & muscularis	Neonatal: 7 days Postnatal: 28 days Adult: 63–168 days	Three main macrophage subsets were identified across all ages: Tim4^+^ CD4^+^ long‐lived resident macrophages, dominant in the neonatal stage (~75%) but reduced to <40% in postnatal and adult stages, and Tim4^−^/CD4^+^ and Tim4^−^ CD4^−^ monocyte‐derived macrophages, which predominated in postnatal and adult stages
Song *et al*.	Mid‐hindgut	Mucosa	Prenatal: E10.5	Embryonically derived Iba‐1^+^ macrophages colonised the mucosa and submucosa of the mid‐hindgut.
Avetisyan *et al*.	Colon & small intestine	Muscularis externa	Prenatal: E9.5–12.5	Iba‐1^+^ macrophages of embryonic origin colonise the outer gut wall by E9.5, coinciding with the formation of the muscularis layers and the initial infiltration of enteric neural crest‐derived cells
Hegarty *et al*.	Colon & small intestine	Mucosa & muscularis	Neonatal: 3 weeks Adult: 12 weeks	Three macrophage subsets were identified: CD11c^+^, CD11c^−^ CD163^−^, and CD163^+^. At week 3, all subsets were present and remained predominantly embryonic‐derived, with no replacement by monocyte‐derived macrophages. By week 12, all subsets were largely replaced by monocyte‐derived macrophages, with only ~3% of CD163^+^ macrophages remaining embryonic‐derived
Chiaranunt *et al*.	Colon	Lamina propria	Adult: 8–12 weeks	Three populations were identified: Tim‐4^−^ CD4^−^ and Tim‐4^−^ CD4^+^ monocyte‐derived macrophages, and Tim‐4^+^ CD4^+^ long‐lived macrophages, the latter were slowly replaced by monocyte‐derived cells

Toll‐like receptors (TLRs) are a family of pattern recognition receptors that detect pathogen‐associated molecular patterns from microbes and activate signalling pathways that initiate innate immune responses and shape adaptive immunity.^24^ In most antigen‐presenting cells, engagement of TLRs triggers strong pro‐inflammatory responses. Intestinal macrophages, however, are a notable exception. While they express multiple TLRs (TLR3‐TLR9), these cells display hypo‐responsiveness to TLR stimulation and generally attenuated pro‐inflammatory cytokine responses, thereby maintaining mucosal defence without inducing tissue‐damaging inflammation.^25^ In prenatal life, this anti‐inflammatory phenotype is shaped by developmental programming. During gestation, intestinal macrophages differentiate under the influence of transforming growth factor (TGFβ2), the expression of which increases as the gut matures.^26^, ^27^ Upon entering the intestinal mucosa, TGFβ2 engages its receptors on macrophages, inducing SMAD2 phosphorylation. This canonical SMAD signalling pathway suppresses pro‐inflammatory cytokines, including tumor necrosis factor (TNF), interleukin 6 (IL6) and IL12, maintaining an anti‐inflammatory state.^26^

Studies using single‐cell RNA sequencing on human foetal tissues have identified highly specialised intestinal macrophage subpopulations defined by homeostatic and clearance functions. In intestinal tissues from post‐conceptional week (PCW) 4–26, Wang *et al*. identified two major macrophage clusters characterised by expression of CD209 and CD207, which were enriched for genes associated with antimicrobial activity (e.g. *LYZ*), DNA clearance (e.g. *DNASE1L3*) and antigen processing and presentation (*CD74*, *HLA class II*).^17^ Another set of observations, examining small and large intestine sections from human foetuses (6–11 PCW),^28^ revealed two dominant macrophage clusters expressing core complement genes *C1QB* and *C1QC* but differing in additional functional markers. One cluster expressed *HLADRB1*, *CCL24*, *IL10RA* and *CLEC10A* genes, suggestive of roles in immune regulation and antigen presentation, whereas the other cluster expressed *LYVE1*, *CD163*, *SPP1* and *LYZ*, indicating involvement in tissue remodelling, and scavenging.^28^ Collectively, findings from these studies highlight the early diversification of intestinal macrophages into functionally distinct subsets that may contribute to both immune surveillance and tissue homeostasis during human foetal development.

It has also been suggested that in addition to a role in immune surveillance, prenatal intestinal macrophages may influence intestinal growth. Using a human‐induced pluripotent stem cell (hiPSC)‐derived macrophage–intestinal organoid co‐culture system, macrophage presence was associated with reduced mesenchymal glycolytic activity and limited organoid expansion following engraftment into immunodeficient mice,^6^ supporting a role for these cells in regulating intestinal metabolism and tissue growth during development.

## Neonatal intestinal macrophages

Early studies using fate‐mapping animal models and flow cytometric analysis of macrophage surface markers examined how intestinal macrophage composition changes after birth.^12^, ^13^ The neonatal period (i.e. the first 2 weeks in mice or initial 28 days in humans) represents a critical phase of immune adaptation, as the newborn intestine is colonised by microbes and develops mechanisms to respond to pathogens.^29^ Two distinct macrophage populations are present: yolk sac‐derived F4/80^hi^ CD11b^lo^ macrophages and haematopoietic stem cell‐derived F4/80^hi^ CD11b^hi^ macrophages.^12^ Although at the early neonatal stage (2 days in mice), F4/80^hi^ CD11b^lo^ macrophages represent 79% of colonic mucosal macrophages, this proportion declines to 43.5% by 3 weeks of age.^12^ By assessing the cell surface markers TIMD4 and CD4 (an apoptotic cell receptor involved in efferocytosis, and a marker of gut macrophage maturation, respectively), Shaw *et al*. demonstrated that TIMD4^+^ CD4^+^ cells constitute the predominant macrophage population in the small intestinal and colonic mucosa of 1‐week‐old mice.^13^ Transcriptomic analysis showed that TIMD4^+^ CD4^+^ macrophages are transcriptionally distinct from blood monocyte‐derived macrophages, and share gene signatures with long‐lived, embryonically derived macrophages (macrophages that are self‐renewing and persist in organs for prolonged periods) such as liver Kupffer cells.^30^ Monocyte‐derived macrophages lacking TIMD4 expression (Table 1) are also present but comprise only a minor proportion of the total macrophage population during the neonatal period.

The anti‐inflammatory state of neonatal intestinal macrophages is shaped by developmental cues and maternal factors.^31^ For example, IL10 limits macrophage activation, while maternal milk components such as β‐casein‐related peptides effectively reduced the percentage of TNF‐positive intestinal macrophages, as well as the expression of CD86 and NOS2, which had been increased by lipopolysaccharide (LPS) stimulation in monocyte‐derived macrophages.^31^, ^32^

In preterm infants, immature intestinal macrophages may retain pro‐inflammatory activity that can contribute to intestinal inflammation.^33^ Intestinal macrophages in preterm human neonates strongly express proinflammatory cytokines such as TNF, IL8 and IL6,^33^, ^34^, ^35^ whereas full‐term neonatal human intestinal macrophages do not.^34^ Several studies underscore the critical role of immature, highly pro‐inflammatory intestinal macrophages in promoting mucosal injury associated with necrotising enterocolitis (NEC), a severe inflammatory bowel disease and a leading cause of mortality in premature neonates.^26^, ^34^, ^36^, ^37^ Using murine models of NEC, researchers have observed an increase in pro‐inflammatory macrophages (e.g. IL1α, TNF, IL8, IL1β and CXCL2‐expressing macrophages) within the Lp of the small and large intestine.^26^, ^37^ An *in vivo* study demonstrated that supplementation with TGFβ2 promoted the maturation of immature, highly pro‐inflammatory intestinal macrophages into non‐inflammatory cells, which in turn protected neonatal mice from NEC‐like intestinal injury.^26^

Intestinal macrophages are thought to play an important role in promoting the proliferation of intestinal endothelial cells during early life.^36^ In the intestine of neonatal mice, CX3CR1^+^ F4/80^+^ CD11b^+^ macrophages and endothelial cells produce substantial amounts of insulin‐like growth factor 1 (IGF1).^36^ *In vivo* studies have shown that macrophage‐derived IGF1 is essential for endothelial cell proliferation within the villus Lp.^36^ Neonatal mice lacking macrophage‐derived IGF1 showed reduced microvasculature development in this region, alongside decreased levels of the proangiogenic factor vascular endothelial growth factor‐A (VEGF‐A), compared to littermate controls.^36^ Importantly, impaired development of the intestinal microvasculature has been linked to NEC.^38^ Mice with IGF1‐deficient macrophages are more susceptible to NEC and treatment with IGF1 decreases intestinal injury in NEC mice.^36^ Additionally, the number of IGF1 positive macrophages and Ki‐67‐positive (proliferative) endothelial cells were significantly lower in tissues of infants with NEC compared to control intestinal tissues from infants undergoing reanastomosis surgery, suggesting that reduced macrophage‐derived IGF1 may impair endothelial cell proliferation and contribute to compromised intestinal vascular integrity in NEC.^36^

## Adult intestinal macrophages

### Ontogeny

Over recent years, increasing attention has been directed towards the heterogeneity of intestinal macrophages in adult tissues. Studies have sought to determine whether distinct macrophage populations occupy defined niches within the different layers of the intestine, exhibit specialised functional roles within these niches, differ in their residence and turnover kinetics and contribute differentially to diseases such as inflammatory bowel disease (IBD). The first evidence of macrophage heterogeneity in the adult murine colon and small intestine,^10^, ^12^, ^13^, ^15^ identified at least two broad populations: monocyte‐derived macrophages and long‐lived macrophages, the latter originally proposed to represent a persistent population derived from embryonic progenitor cells. A range of markers, including CD4, TIMD4, CCR2, CD206, CD169 and CD11b, have been used to distinguish between these populations.^10^, ^12^, ^13^, ^15^, ^39^ However, recent studies suggest that while intestinal macrophages are predominantly replenished by monocytes, this process is highly dependent on the anatomical niche and subset. Evidence also indicates that a proportion of embryonically derived macrophages can persist long term within specific niches, resulting in distinct replenishment dynamics across different macrophage subsets.^10^, ^39^, ^40^

Monocyte‐derived intestinal macrophages are maintained through CCR2‐dependent recruitment of classical Ly6C^hi^ monocytes.^12^, ^13^ This process is described by the waterfall model, in which Ly6C^hi^ CX3CR1^int^ blood monocytes enter the intestine and undergo stepwise differentiation into resident macrophages through defined intermediate stages (P1–P4).^12^, ^13^ During this transition, monocytes progressively downregulate Ly6C and upregulate MHCII, ultimately giving rise to mature intestinal macrophages. The final stages comprise MHCII^hi^ macrophages that can be subdivided into CX3CR1^int^ (P3) and CX3CR1^hi^ (P4) populations, with CX3CR1^hi^ cells representing the most mature resident macrophages.^12^, ^13^ Hegarty *et al*. used single‐cell transcriptomic analysis to identify three macrophage clusters in the murine colon. One cluster expressed *Itgax* and matrix metalloproteinases, consistent with tissue‐remodelling functions; a second expressed *Cd163*, *Folr2*, *Lyve1* and genes associated with complement activation and phagocytosis; and a third lacked *Itgax* and *Cd163* and was enriched for genes involved in macrophage‐fibroblast interactions.^40^ These clusters corresponded to CD11c^+^, CD163^+^ and CD11c^−^/CD163^−^ populations, with CD163^+^ macrophages predominantly located in the deeper mucosa and submucosa, closely associated with blood vessels and nerves, while CD11c^+^ macrophages were mainly confined to the mucosal layer.^40^ This transcriptional and functional heterogeneity has also been reported in other studies conducted in both mice^10^, ^39^ and humans.^7^, ^28^, ^41^ Analysis of adult human intestinal macrophages showed a differentiation trajectory (termed Mf1–Mf4 stages), which closely mirrors the murine ‘waterfall’ model, emphasising the diverse functional roles of these macrophages within the intestinal microenvironment.^41^ Under steady‐state conditions, Bujko *et al*. demonstrated that small intestinal macrophages are derived from peripheral blood monocytes and progress through intermediate stages: Mf1 (CD14^+^ CD11c^+^ HLA‐DR^int^) and Mf2 (CD14^+^ CD11c^+^ HLA‐DR^hi^), before maturing into Mf3 (CD14^+^ CD11c^−^ CD11b^−^ HLADR^hi^) and Mf4 (CD14^hi^ CD11c^−^ CD11b^+^ HLADR^hi^) subsets.^41^ The Mf3 subset constitutes most macrophages within intestinal tissues (~61%) and is primarily located in the villous mucosa of the small intestine. In contrast, CD11b^+^ Mf4 macrophages are predominantly located in the deeper submucosa and muscularis propria, where they express Bone Morphogenetic Protein 2 (BMP2), suggesting they may be homologous to mouse muscularis macrophages (MMs) and could play roles related to the enteric nervous system (ENS) and intestinal motility.^41^ Moreover, using human intestinal tissues from the small intestine and colon, analyses showed that macrophages can be divided into several distinct transcriptional clusters.^7^, ^41^ Some clusters were enriched for genes associated with canonical immune functions, including responses to bacteria and fungi, as well as antigen processing and presentation. These clusters showed high expression of genes such as *S100A8–9*, *IL10*, *CXCL2*, *GBP1–5, CXCL8–11, CD1C* and *CD1E*. Other clusters expressed genes characteristic of long‐lived macrophages, including *LYVE1*, *COLEC12*, *F13A1* and *FOLR2*, and were associated with biological functions such as synapse pruning, clearance of apoptotic cells, chemotaxis and tissue‐protective properties.^7^, ^41^ These long‐lived macrophages were primarily located in the submucosa and resemble the CD163^+^ macrophages identified in murine colon tissue, whereas clusters associated with canonical immune functions were mainly found in the subepithelial regions of the intestine, consistent with findings reported in murine studies.^40^

**Figure 2:**
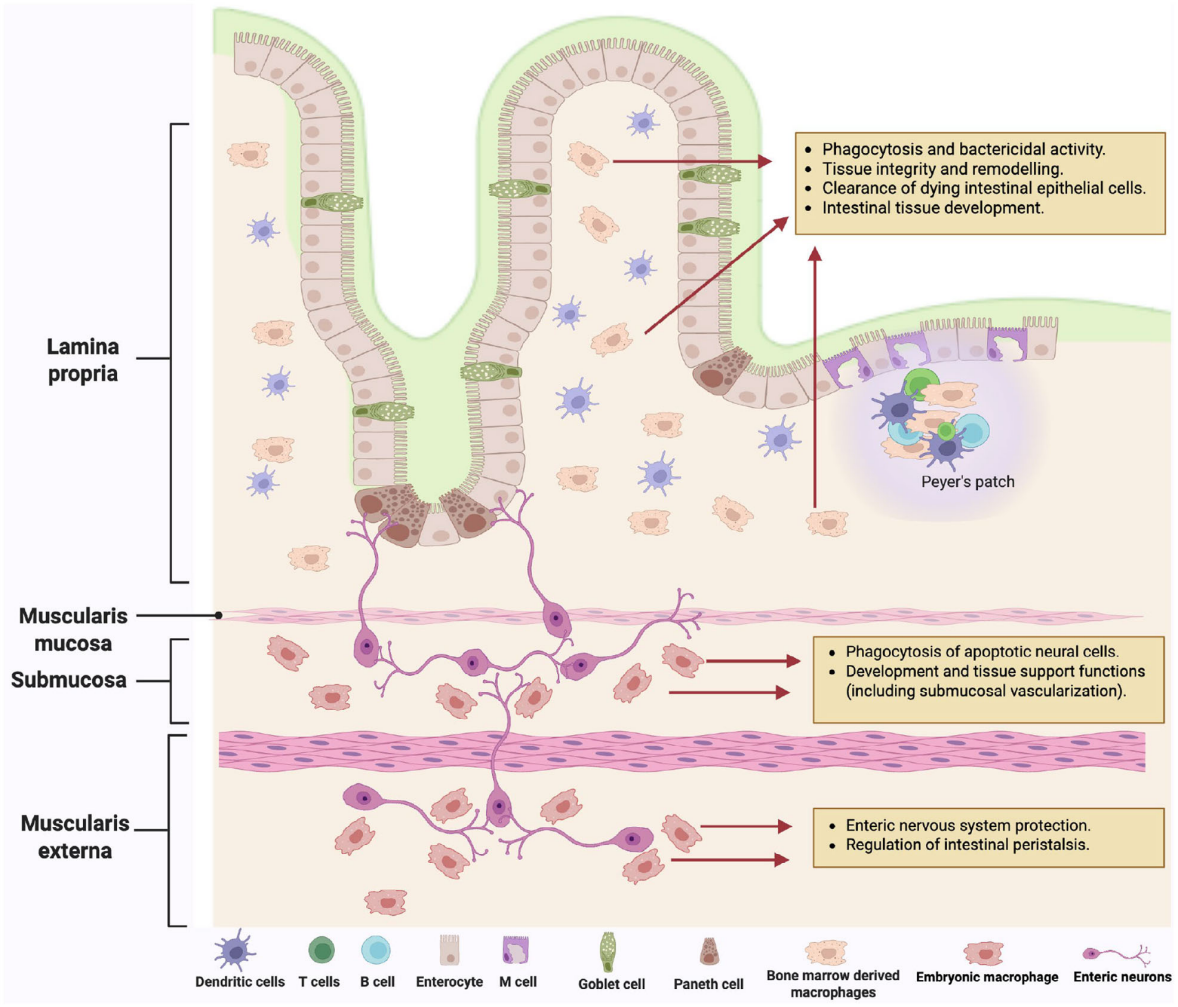
Schematic indicating known function and localisation of macrophages within histological layers of the intestine. Intestinal macrophages are heterogeneous and can be divided into two populations based on their developmental origin: bone marrow‐derived macrophages (BMDMs) and embryonically derived macrophages (EDMs). In mice, BMDMs are characterised by expressions of MHC‐II^+^, CD64^+^, CD206^+^, CD163^+^ and TIM‐4^−^/CD4^+/−^. In humans, BMDMs express CD4^+^ and CD209^+^, contributing to tissue integrity and remodelling in addition to other functions. In mice, EDMs are characterised by MHC‐II^+^, CD64^+^, CD206^+^, CD163^+^, TIM‐4^+^, CD4^+^, CD11b^+^, and CD11c^−^. In humans, EDMs are characterised by MHC‐II^+^, CD64^+^, CD206^+^, CD163^+^, and CD209^+^, contributing to enteric nervous system protection alongside other functions. Created with BioRender.com, Toronto, Canada.

Importantly, emerging data indicate that all adult intestinal macrophage subsets are replenished by circulating blood monocytes at distinct, subset‐specific rates. Even long‐lived macrophages, which have been associated with embryonic origin, undergo progressive monocyte‐dependent replacement. Embryonic progenitors nonetheless make a limited contribution across multiple macrophage subsets rather than being confined to a single marker‐defined population. Lineage‐tracing studies using *Ccr2* ^CreERT2^ × *Rosa26*‐LSL‐tdTomato mice demonstrated that ~17% of TIMD4^+^ colonic macrophages were replaced by monocyte‐derived cells within 1 week, a substantially slower turnover compared with TIMD4^−^/CD4^−^ and TIMD4^−^/CD4^+^ macrophages, which showed replacement rates exceeding 60% in a week.^39^ Consistent with these findings, Hegarty *et al*. showed that all small intestine and colon macrophage subsets of adult mice undergo monocyte‐mediated replenishment, with higher replacement rates in CD11c^+^ and CD11c^−^/CD163^−^ macrophages, whereas CD163^+^ macrophages exhibited significantly slower turnover.^40^ Fate‐mapping using *Ms4a3* ^Cre/+^ *Rosa26‐* CAG‐LSL‐tdTomato mice further revealed that intestinal macrophages are predominantly embryonically derived at 3 weeks of age, with a marked increase in monocyte contribution by 12 weeks, reflecting postnatal recruitment of haematopoietic‐derived monocytes. However, a small fraction of embryonically derived macrophages persisted across subsets during adulthood; this contribution declined progressively, and by 1 year of age all intestinal macrophages were of monocyte origin.^40^ While these data support the presence of long‐lived embryonically derived macrophages in adult murine samples under 1 year, they also show that these cells are found across all macrophage subsets rather than in distinct subpopulations (Figure 2).

### Factors that maintain macrophage maturation and colonisation

While the factors that mediate differences in macrophage longevity among intestinal macrophage subsets remain unknown, it is important to highlight that, in general, the replenishment, colonisation and maturation of adult intestinal macrophages are controlled by a combination of molecular and environmental factors.

#### CSF1R signalling

CSF1R signalling, mediated by its ligands, CSF1 and IL34, is essential for macrophage proliferation, differentiation and survival.^18^, ^23^, ^42^ Genetic disruption of either CSF1 or its receptor in mice results in a striking deficiency of macrophages across multiple tissues, underscoring the central role of this axis in macrophage biology. In adult mice, pharmacological inhibition of CSF1R, either with an antibody or the small‐molecule inhibitor pexidartinib, results in a marked reduction of macrophages in the intestinal mucosa^23^, ^43^ and near‐complete depletion of MMs.^18^, ^42^ However, the main source of the CSF1 in intestinal tissues remains incompletely identified. A recent study reported that Podoplanin‐positive (PDPN^+^) fibroblasts, a dominant stromal population, are the main producers of CSF1 in the Lp of both the small and large intestine.^23^ The data from this study indicate spatial specialisation in macrophage dependence on CSF1: upper Lp macrophages are maintained cooperatively by fibroblast‐derived CSF1 and IL34, whereas macrophages in the lower Lp and submucosa rely predominantly on fibroblast‐derived CSF1, with a lesser contribution from endothelial‐derived CSF1.^23^ In line with the findings of Hegarty *et al*., Nonaka *et al*. further demonstrated an accumulation of CD163^+^ macrophages within the submucosa and revealed that their abundance is tightly regulated by CSF1 derived from locally CD81^+^ LepR^+^ fibroblasts.^23^, ^40^ The role of epithelial cells appears most critical during early development.^22^ Evidence indicates that intestinal epithelial cells (IECs) are a dominant source of CSF1 in the neonatal gut, where their secretion is essential for establishing the initial resident macrophage pool; a downregulation of epithelial‐derived CSF1 is a hallmark of inflammatory conditions such as NEC, leading to a loss of homeostatic niche support.^22^ Moreover, consistent with findings from fibroblast‐derived IL34, genetic ablation of epithelial IL34 in adult murine models does not significantly affect the overall abundance of macrophages within the intestinal mucosa, but instead influences macrophage polarisation, promoting a shift towards an anti‐inflammatory phenotype, particularly under pathological conditions.^44^ This effect is especially evident in gastrointestinal graft‐versus‐host disease (GI GVHD), a major adverse outcome following allogeneic haematopoietic stem cell transplantation (HSCT), characterised by dysregulated interactions between effector and regulatory immune compartments. In this setting, IL34 supports intestinal epithelial cell survival and contributes to the maintenance of mucosal barrier integrity.^44^

Finally, endothelial cells provide a secondary but essential layer of support, particularly for macrophages residing in the lower villus and submucosa.^23^ Evidence suggests that while fibroblast‐derived signals cover the broad mucosal surface, endothelial‐derived CSF1 maintains a separate, vasculature‐associated macrophage population that is functionally distinct from those supported by the epithelium or fibroblast CSF1.^23^

While CSF1R signalling is fundamental for the steady‐state colonisation and maintenance of the intestinal macrophage pool, Granulocyte–macrophage colony‐stimulating factor (GMCSF, also known as CSF2) functions as a key regulator of mucosal macrophage activity during inflammation and tissue injury. Early studies demonstrated that CSF2‐deficient mice develop more severe acute colitis, associated with impaired macrophage recruitment and weakened innate immune responses to bacterial invasion.^45^ More recent work has refined this understanding by identifying specific cellular sources and downstream effects on macrophage differentiation. Tomas and colleagues showed that Group 3 innate lymphoid cells (ILC3s) are a major inducible source of CSF2 during inflammation, and that ILC3‐derived CSF2 is required for the full maturation of monocytes into P3 and P4 subsets.^46^ In its absence, immature monocytes accumulate and exhibit reduced antimicrobial function.^46^ Complementary findings from a 2024 study further demonstrated that the intestinal stroma, particularly fibroblasts, directs monocyte‐to‐macrophage differentiation through CSF2 secretion.^47^ Notably, this work indicates that while CSF2 promotes a protective pro‐inflammatory state, it concurrently suppresses wound‐healing programmes. In the absence of CSF2, macrophages adopt a pro‐repair phenotype characterised by increased expression of collagen and PDGFBB, which may contribute to pathological intestinal fibrosis and stricture formation in Crohn's disease.

Muscularis macrophages are resident cells located between the circular and longitudinal muscle layers of the gut. Closely associated with myenteric neurons, they are distinct from mucosal macrophages in structure, gene expression and function.^48^, ^49^ Studies suggested that enteric neurons are the main source of CSF1 in the adult gut.^42^ However, during gut development, CSF1 in the muscularis is largely produced by non‐neuronal sources, including endothelial cells and interstitial cells of Cajal (ICCs), with some data showing enteric neurons contributing but not exclusively to CSF1 production in adult gut tissues.^18^ Moreover, human and murine models lacking enteric neurons show no change in MM numbers and activity, indicating that neuronal CSF1 is not essential for their colonisation or function.^18^

#### IL10/IL10R signalling

IL10/IL10R is a central regulator of inflammatory homeostasis in mature intestinal macrophages, ensuring that immune responses in the gut mucosa remain controlled despite constant microbial exposure.^50^, ^51^ Intestinal macrophages respond to IL10 through a heterodimeric receptor complex composed of the ligand‐binding subunit IL10Rα and the shared accessory subunit IL10Rβ.^52^ Upon ligand engagement, the receptor complex initiates signal transducer and activator of transcription 3 (STAT3) phosphorylation, leading to its dimerisation and translocation into the nucleus.^52^, ^53^ Nuclear STAT3 drives a regulatory transcriptional programme characterised by the induction of suppressive molecules such as SOCS3, ETV3 and BCL3. These factors collectively repress the transcription of pro‐inflammatory mediators, including TNF, IFNγ, IL6, IL23 and IL12, thereby maintaining macrophages in a controlled, non‐pathogenic state.^52^, ^54^, ^55^, ^56^ Disruption of this signalling axis has significant pathological consequences. In murine models, ablation of IL10R or STAT3 in intestinal macrophages results in uncontrolled production of IL23 and chemokines such as CCL3 and CCL5 by mucosal macrophages.^54^, ^55^, ^57^ This promotes excessive recruitment of inflammatory monocytes and drives IL22‐dependent epithelial inflammatory responses, ultimately leading to spontaneous and severe colitis.^58^, ^59^ In humans, loss‐of‐function mutations in *IL10*, *IL10RA* or *IL10RB* cause very‐early‐onset IBD with severe clinical phenotypes, highlighting the essential role of IL10‐mediated signalling in preserving intestinal immune equilibrium.^60^

#### Gut microbiota

The gut microbiota, predominantly composed of the *Bacillota* (*Firmicutes*) and *Bacteroidetes* phyla,^61^ has been shown to regulate both the abundance and functional programming of intestinal macrophages. In the absence of these microorganisms, as observed in germ‐free (GF) mice, the numbers of monocytes, monocyte‐derived macrophages and long‐lived tissue‐resident (embryonically derived) intestinal macrophages are reduced.^12^, ^13^, ^14^, ^62^ Data from Chen *et al*. demonstrated that Ly6C^hi^ monocytes, monocyte‐derived macrophages (Ly6C^+^MHCII^+^ and Ly6C^−^MHCII^+^) and F4/80^hi^ long‐lived resident macrophages are all significantly reduced in the mucosa of both the colon and small intestine in GF mice compared with specific pathogen‐free (SPF) controls.^14^ The extent to which the microbiota influences macrophage turnover kinetics remains incompletely understood. While earlier work by Bain *et al*.^12^ suggested that a reduction in colonic macrophages might stem from impaired monocyte replenishment in the absence of microbial signals, a subsequent study using fate‐mapping models reached a more nuanced conclusion.^14^ Specifically, Chen *et al*. demonstrated that, unlike F4/80^hi^ resident macrophages, the replacement kinetics of monocyte‐derived macrophages in the colon are comparable between GF and SFM mice under steady‐state conditions.^14^ These findings indicate that while microbiota may set the overall size of the macrophage pool, the actual rate at which monocyte‐derived cells are replenished by bone marrow precursors is a process that remains largely independent of commensal microbes.

The impact of the microbiota on key factors involved in maintaining the intestinal macrophage population has also been investigated. Expression of Csf1, along with inflammatory cytokine genes such as Tnf and Il1β, is significantly reduced in both the colon and small intestine of GF.^14^ In addition, microbiota‐derived metabolites, including short‐chain fatty acids (SCFAs), act as histone deacetylase (HDAC) inhibitors that epigenetically suppress pro‐inflammatory cytokine production.^63^ Exposure of colonic Lp macrophages to n‐butyrate, one type of SCFA, reduces the production of pro‐inflammatory mediators, including nitric oxide, IL6 and IL12, via HDAC inhibition.^63^ SCFAs following antibiotic treatment render intestinal macrophages more responsive to LPS, leading to markedly increased production of inflammatory cytokines and a sustained enhancement of Th1 cell responses.^64^ Other bacterial products have also been shown to influence intestinal macrophage polarisation and are reviewed in.^65^ While this section does not encompass all studies examining the relationship between microbiota and intestinal macrophage function and maintenance, the collective evidence highlights a central role for commensal microbes in shaping macrophage abundance, phenotype, and inflammatory responsiveness. Several other factors, including epithelium‐derived signals and transcription factors such as ZEB2, RUNX3 and NR4A1, have also been shown to contribute to the differentiation and maturation of intestinal macrophages. These factors are discussed elsewhere.^66^

## Immune and non‐immune functions of intestinal macrophages in health

In the healthy intestinal mucosa, the Lp macrophages, specifically the CX3CR1^hi^ resident population, act as the primary sentinels for maintaining gut homeostasis. Under normal conditions, these macrophages are highly phagocytic and actively sample the environment by projecting transepithelial dendrites between intestinal epithelial cells into the gut lumen.^67^ This mechanism, facilitated by the CX3CL1‐CX3CR1 axis, allows them to capture and clear pathogens and their derivatives without compromising the integrity of the epithelial barrier.^67^ Efferocytosis, defined as the phagocytic clearance of apoptotic cells, represents a substantial homeostatic demand within the intestinal mucosa, where billions of epithelial cells are turned over daily.^5^ Resident Lp macrophages, characterised by high expression of Mer tyrosine kinase (Mertk) and the CX3CR1, are central mediators of this process.^5^ These cells recognise canonical ‘Eat‐Me’ signals, particularly externalised phosphatidylserine on apoptotic cells, enabling rapid engulfment prior to secondary necrosis and the release of damage‐associated molecular patterns.^5^ Beyond clearing apoptotic cells, mucosal macrophages secrete a range of cytokines and chemokines that help regulate overall intestinal immune homeostasis.^2^ In addition to IL10, resident Lp macrophages produce basal levels of IL1β in response to commensal microbiota, supporting the maintenance of T helper 17 (Th17) cells, which are essential for mucosal defence.^2^ Macrophages also serve as key sources of chemokines such as CCL2, CCL8, CXCL1 and CXCL2, promoting the recruitment of neutrophils, monocytes and T cells, further contributing to intestinal homeostasis.^68^ Moreover, mucosal macrophages contribute to structural and functional maintenance of the mucosal niche. They regulate extracellular matrix turnover through the controlled secretion and endocytic recycling of matrix metalloproteinases,^69^ thereby preserving Lp integrity while maintaining tissue compliance required for peristalsis. Additional evidence indicates that these functions extend to active regulation of the epithelial niche. Intestinal macrophages maintain essential WNT signalling required for intestinal stem cell maintenance and proliferation,^70^ thereby directly supporting epithelial renewal. They also participate in transmitophagy, a process involving the phagocytic acquisition of stressed or dysfunctional mitochondria from epithelial cells, which promotes epithelial metabolic homeostasis and cellular fitness.^71^

Muscularis macrophages act as regulators of gastrointestinal motility and protectors of the ENS.^72^ They are strategically located within the myenteric and submucosal plexuses.^42^, ^72^ Several lines of evidence suggest that the ENS can directly communicate with MMs. While some studies showed MMs form synapses with enteric neurons, Muller *et al*. reported that MMs produce BMP2 to support neuronal development and survival.^42^ Beyond motility, MMs provide neuroprotective and trophic support through the expression of heme oxygenase 1 (HO1) and the secretion of prostaglandin E2 (PGE2), protecting both the ENS and ICCs from oxidative stress and preserving intestinal wall integrity.^10^ Recent evidence also shows that MMs contribute to ENS homeostasis by releasing CIQ, which regulates neuronal gene expression and prevents excessive intestinal transit.^73^ Interesting data came from Gabanyi *et al*. showing that MMs preferentially express tissue‐protective and wound‐healing genes, including *Retnla*, *Mrc1*, *Cd163* and *Il10*, as well as higher CD86 expression, compared with Lp macrophages.^74^ They found that MMs exhibit a more anti‐inflammatory profile overall than Lp macrophages. Mechanistically, MMs express higher levels of β2‐adrenergic receptor (β2AR), enabling them to respond to catecholamines such as noradrenaline released by enteric neurons. Deletion of β2AR in macrophages disrupts their homeostatic gene programme and impairs epithelial repair following intestinal injury, indicating that adrenergic signalling is required for tissue‐protective macrophage programming.^74^ However, all these functions have been described in the context of the broader intestinal macrophage population, which is highly heterogeneous and comprises multiple transcriptionally distinct subsets. Expanding analyses to examine the specific functions associated with each subset will improve our understanding of their individual contributions to intestinal physiology and pathology, including their roles in disease development and tissue homeostasis.

## Macrophages in IBD

Inflammatory bowel disease is defined as a group of chronic, immune‐mediated inflammatory disorders affecting the gastrointestinal tract, including Crohn's disease and ulcerative colitis.^75^ Globally, IBD affects approximately 7 million individuals as of 2025, with a pooled incidence of around 10 cases per 100 000 person‐years.^76^ The pathogenesis of IBD is complex and multifactorial but is one example of a macrophage‐regulated disorder, involving an inappropriate immune response of the gut in genetically susceptible individuals, triggered by environmental factors.^75^, ^77^ Multiple intestinal cell populations participate in IBD pathogenesis, including epithelial, innate and adaptive immune, stromal and endothelial cells. Intestinal epithelial cells preserve barrier integrity via tight junction proteins, including claudins and occludin, with disruption increasing permeability and microbial translocation.^78^ Genetic variations in genes such as *CLDN2*, *OCLN*, or *MAGI2* can compromise tight junction formation, resulting in barrier defects.^79^, ^80^ Goblet cells produce MUC2, the principal mucin forming the protective mucus layer, which is reduced or structurally altered in ulcerative colitis.^81^ Paneth cells secrete antimicrobial peptides including α‐defensins; genetic variants in *NOD2* and *ATG16L1* impair antimicrobial function and autophagy, linking epithelial innate defence defects to Crohn's disease susceptibility.^82^ Dendritic cells in the Lp detect antigens and shape T‐cell responses. In IBD, their overproduction of IL12 and IL23 drives pro‐inflammatory Th1 and Th17 cells,^83^ creating an environment of high IFNG, TNF, IL17 and IL22. This inflammatory milieu overwhelms Treg cells, limiting their ability to suppress inflammation.^84^ B cells increase IgG responses to microbial antigens,^85^ and neutrophils exacerbate tissue injury via reactive oxygen species and proteases.^86^ ILC3s contribute to both epithelial repair (IL22) and inflammation (IL17).^87^ Stromal fibroblasts promote fibrosis, and endothelial ICAM1 and VCAM1 expression supports persistent leukocyte recruitment.^88^

Alongside the above‐mentioned cell types in IBD, macrophages are also understood to play a critical role in inflammatory exacerbation. Consistent with this, many IBD susceptibility loci are linked to monocyte and macrophage function. These include genes involved in pathogen recognition and autophagy, such as *NOD2*, *IRGM*, *LRRK2*, *XBP1* and *ATG16L1*, as well as regulators of macrophage differentiation and function, including *IL10*, *IL13RA*, *FOSL2*, *PTGER4* and *TNFAIP3*.^60^, ^89^, ^90^, ^91^, ^92^, ^93^, ^94^, ^95^ IBD is characterised by a profound shift in intestinal macrophage populations, marked by the accumulation of pro‐inflammatory monocyte‐derived macrophages and a concomitant reduction in tolerogenic resident macrophages. Under inflammatory conditions, circulating CCR2^+^ CD14^+^ monocytes are actively recruited into the intestinal mucosa, where they differentiate into immature CD14^+^ CD64^+^ CCR2^+^ CX3CR1^int^ macrophages that produce high levels of inflammatory mediators, including TNF, IL1β and IL6, thereby driving intestinal inflammation and tissue injury.^96^ These inflammatory macrophages exhibit reduced expression of resident macrophage markers such as CD163 and CD206, reflecting incomplete maturation and impaired acquisition of regulatory and tissue‐protective functions.^41^, ^54^, ^97^ In contrast, homeostatic intestinal macrophages, defined by high expression of CX3CR1, CD163, CD206, Mertk and complement components such as C1QC, represent fully differentiated tissue‐resident macrophages.^54^, ^97^ In IBD, these resident CX3CR1^high^ CD163^+^ CD206^+^ C1QC^+^ macrophages are markedly diminished or functionally displaced because of continuous recruitment of inflammatory monocytes and disruption of the normal monocyte‐to‐macrophage differentiation trajectory.^98^, ^99^ Single‐cell RNA sequencing has further refined this model by identifying *S100A8* ^+^ *S100A9* ^+^ *IL1β* ^+^ macrophages and *SPP1* ^+^ macrophages, which exhibit hyper‐inflammatory and profibrotic transcriptional programmes and are associated with chronic inflammation and resistance to anti‐TNF therapy.^98^ Additionally, specialised resident subsets such as LYVE1^+^ macrophages, which contribute to tissue repair and vascular homeostasis, are reduced during intestinal inflammation, further impairing mucosal healing.^10^ The transition towards a pro‐inflammatory macrophage phenotype can compromise mucosal integrity by driving elevated metalloproteinase release, particularly MMP9. This leads to degradation of the extracellular matrix, increased intestinal permeability, and enhanced recruitment of additional immune cells.^100^ Several factors have been implicated in driving a shift in the macrophage milieu towards a pro‐inflammatory state. Notably, deficiencies in key regulatory pathways, including IL10, IL10 receptor and TGFβ signalling, have been well‐documented in patients with IBD.^101^, ^102^ In addition, patients with IBD frequently demonstrate elevated expression of SMAD7, an intracellular inhibitor of TGFβ1 signalling, which prevents this cytokine from exerting its anti‐inflammatory effects.^102^ This impairment promotes excessive production of pro‐inflammatory cytokines, including IFNG, IL6 and IL1β, which are consistently increased in the intestinal mucosa of IBD patients and contribute to epithelial barrier damage.^101^, ^102^ IL6 drives pathological changes in the intestinal mucosa by enhancing tissue swelling, compromising epithelial barrier integrity and engaging NF‐κB pathways through STAT3 activation, which together disturb cytokine balance and worsen tissue injury in ulcerative colitis.^103^ Importantly, IL1β has emerged as a therapeutic target, as treatment with IL1 receptor antagonists such as Anakinra has shown potential in reducing systemic inflammation in patients with IL10 receptor deficiency.^101^ Moreover, in the context of IBD therapies and beyond IL10‐based approaches,^104^ strategies aim to enhance anti‐inflammatory macrophage responses while limiting pro‐inflammatory activity, with certain pharmacological agents exemplifying this approach. Thalidomide, for instance, suppresses pro‐inflammatory macrophage polarisation by inhibiting the transcription factor IRF5, leading to reduced production of inflammatory mediators, including IL12 and IFNG, and supporting repair of the intestinal mucosa.^105^ In parallel, paroxetine has been reported to attenuate inflammatory activity in ulcerative colitis by disrupting the interaction between G protein‐coupled receptor kinase 2 (GRK2) and prostaglandin E2 receptor 4 (EP4), which lowers intracellular cAMP levels and promotes anti‐inflammatory macrophage polarisation.^106^

CSF2, as noted above, has also been identified as a key regulator of macrophage activation during intestinal macrophages. In murine colon, ILC3s are the principal source of CSF2, and depletion of ILCs induces tissue‐reparative transcriptional programmes in macrophages, indicating that ILC3‐derived CSF2 promotes inflammatory macrophage activation while restraining pro‐repair, wound‐healing phenotypes.^46^ Although reparative responses are essential for barrier restoration, their dysregulation contributes to fibrosis and intestinal stricturing, a major complication of inflammatory bowel disease.^46^ These findings suggest that ILC3‐derived CSF2 may limit fibrotic progression by modulating macrophage polarisation. Single‐cell RNA sequencing has highlighted other genes such as *RUNX3*, *IL21R*, *GTF2I* and *LILRB3* that are associated with the failure of monocytes to transition into mature, protective macrophages.^107^

Metabolic reprogramming is also appreciated as a fundamental mechanism controlling macrophage pathogenicity in IBD. Unlike resident macrophages that rely on oxidative phosphorylation (OXPHOS) for energy, inflamed IBD macrophages switch to aerobic glycolysis to meet their high‐energy demands for cytokine production.^108^, ^109^ This metabolic shift is driven by the transcription factor Hypoxia‐Inducible Factor 1 alpha (HIF1α) and the accumulation of the metabolite succinate, which stabilises HIF1α and directly promotes the transcription of IL1β.^109^ Additionally, these macrophages exhibit disruptions in Krebs cycle pathways and impaired fatty acid oxidation, which further locks them into a pro‐inflammatory state.^108^, ^110^ Overall, it has been suggested that targeting these metabolic pathways, for instance, by promoting OXPHOS or inhibiting glycolysis, could re‐functionalise macrophages back to a healing phenotype, offering a novel therapeutic avenue beyond standard anti‐inflammatory drugs. Indeed, a recent study reported an engineered nanozyme (PPFeCs) that reduces DSS‐induced colonic inflammation and improves epithelial barrier function. It acts by shifting macrophage metabolism from glycolysis to oxidative phosphorylation via PI3K/Akt modulation and inhibiting NFκB signalling, promoting a switch from pro‐inflammatory to anti‐inflammatory macrophages and dampening inflammation.^111^

## Intestinal macrophages during aging

Although numerous studies have investigated the effects of ageing on the intestinal mucosal barrier, immune populations such as dendritic cells and T cells, and gut microbiome composition reviewed in,^112^, ^113^, ^114^ direct evidence on how ageing impacts intestinal macrophages remains relatively limited. Current evidence indicates that macrophages within the muscularis externa undergo marked phenotypic changes with age, shifting from an anti‐inflammatory to a pro‐inflammatory state.^115^ This transition is characterised by increased production of pro‐inflammatory cytokines, including IL1β, IL6 and TNF.^115^ Although the mechanisms driving this age‐associated shift in macrophage cytokine expression are not yet fully understood, available data suggest that the acquisition of a pro‐inflammatory phenotype contributes to disruption of intestinal homeostasis and so this is a mal‐adaptive response. In aged mice, increased numbers of pro‐inflammatory macrophages have been associated with enhanced apoptosis of enteric neurons and prolonged intestinal transit time compared with younger animals, indicating impaired gut function.^115^ As yet, there is little to no evidence on the effects of aging on the phenotype and function of Lp macrophages or on how age‐associated changes in the gut microbiota influence intestinal macrophage biology. These factors remain to be studied.

## Conclusion and future insights

This review highlights the dynamic ontogeny, heterogeneity and functional specialisation of intestinal macrophages across the lifespan from prenatal developmental through to aging. We have demonstrated these cells arise from distinct developmental origins and diversify into specialised subsets shaped by local niche signals and microbial exposure. Recent advances in single‐cell and spatial technologies have revealed a level of complexity far exceeding previous models, emphasising the need to reinterpret earlier functional observations in the context of defined macrophage subsets. Despite these advancements, several critical gaps remain. Although emerging evidence underscores the critical roles of macrophages in embryonic intestinal growth, further investigation is needed to understand specific contributions at various stages of intestinal development. Similarly, the exact mechanisms by which the microbiota regulate macrophage turnover kinetics and subset identity are not yet fully defined. Furthermore, reconciling murine models with human intestinal biology remains a priority, particularly regarding how these findings apply to clinical disease states. The impact of biological sex also remains largely unexplored. Given the known sex‐based disparities in inflammatory diseases, understanding how sex hormones and genetic factors influence macrophage ontogeny is essential for developing inclusive models of immunity. Ageing represents another under‐investigated dimension; while senescence appears to drive pro‐inflammatory shifts in the muscularis externa that impair gut motility, the impact of age on lamina propria macrophages and their interaction with the microbiome remains poorly understood. In the end, resolving these questions will deepen our understanding of intestinal macrophage biology, establishing the essential framework for developing targeted therapies that modulate macrophage function to combat inflammatory disorders and restore gut homeostasis.

## Search strategy

This is a narrative review. Data for this review were identified by searches of PubMed, Google Scholar and Web of Science. Search terms included, but were not limited to, intestinal macrophages, ontogeny, prenatal gut immunity, foetal intestinal macrophages, monocyte waterfall, MMs, inflammatory bowel disease and gut microbiota. Articles were selected based on their relevance to macrophage development, niche‐specific functions, and homeostatic regulation within the gastrointestinal tract. Priority was given to high‐impact original research. Only articles published in English were included. Review articles were screened to identify additional primary sources.

## Author contributions

**Elisa L Hill‐Yardin:** Conceptualization; supervision; validation; visualization; writing – original draft; writing – review and editing. **Sarah J Spencer:** Conceptualization; supervision; visualization; writing – original draft; writing – review and editing; validation; funding acquisition. **Baha Mustafa:** Conceptualization; writing – original draft; writing – review and editing; visualization; validation.

## Conflict of Interest

The authors declare no competing interests except that Prof. Hill‐Yardin is a scientific advisor for the prebiotics company, Adepa (Australia).

## Data Availability

Data sharing not applicable to this article as no datasets were generated or analysed during the current study.
